# National Trends over One Decade in Hospitalization for Acute Myocardial Infarction among Spanish Adults with Type 2 Diabetes: Cumulative Incidence, Outcomes and Use of Percutaneous Coronary Intervention

**DOI:** 10.1371/journal.pone.0085697

**Published:** 2014-01-15

**Authors:** Ana Lopez-de-Andres, Rodrigo Jimenez-Garcia, Valentin Hernandez-Barrera, Isabel Jimenez-Trujillo, Carmen Gallardo-Pino, Angel Gil de Miguel, Pilar Carrasco-Garrido

**Affiliations:** 1 Preventive Medicine and Public Health Department. Rey Juan Carlos University. Alcorcón, Madrid, Spain; University of Verona, Ospedale Civile Maggiore, Italy

## Abstract

**Background:**

This study aims to describe trends in the rate of acute myocardial infarction (AMI) and use of percutaneous coronary interventions (PCI) in patients with and without type 2 diabetes in Spain, 2001–2010.

**Methods:**

We selected all patients with a discharge of AMI using national hospital discharge data. Discharges were grouped by diabetes status: type 2 diabetes and no diabetes. In both groups PCIs were identified. The cumulative incidence of discharges attributed to AMI were calculated overall and stratified by diabetes status and year. We calculated length of stay and in-hospital mortality (IHM). Use of PCI was calculated stratified by diabetes status. Multivariate analysis was adjusted by age, sex, year and comorbidity. Results: From 2001 to 2010, 513,517 discharges with AMI were identified (30.3% with type 2 diabetes). The cumulative incidence of discharges due to AMI in diabetics patients increased (56.3 in 2001 to 71 cases per 100,000 in 2004), then decreased to 61.9 in 2010. Diabetic patients had significantly higher IHM (OR, 1.14; 95%CI, 1.05–1.17). The proportion of diabetic patients that underwent PCI increased from 11.9% in 2001 to 41.6% in 2010. Adjusted incidence of discharge in patients with diabetes who underwent PCI increased significantly (IRR, 3.49; 95%CI, 3.30–3.69). The IHM among diabetics patients who underwent a PCI did not change significantly over time.

**Conclusions:**

AMI hospitalization rates increased initially but declining slowly. From 2001 to 2010 the proportion of diabetic patients who undergo a PCI increased almost four-fold. Older age and more comorbidity may explain why IHM did not improve after a PCI.

## Introduction

Diabetes is a major risk factor for atherosclerosis, which predisposes patients to occlusive coronary artery disease (CAD), acute myocardial infarction (AMI), and death [1]. It is well established that the long-term prognosis of AMI is worse in patients with diabetes than in those without diabetes [2], [3]. In fact, the mortality rate for AMI is approximately double in patients with diabetes [3].

Patients with diabetes are prone to a diffuse and rapidly progressive form of CAD, which increases their likelihood of undergoing revascularization procedures [4]. Approximately one-third of all percutaneous coronary interventions (PCI) performed each year in the US are in patients with diabetes [5]. As the prevalence of diabetes increases, the number of patients with diabetes requiring revascularization for advanced CAD will escalate [6]. Although management of patients with CAD has improved considerably, coronary event rates remain very frequent, and mortality is greater among patients with diabetes [7].

Secular trends in the use of PCI in patients with diabetes have been examined [8], [9]. In the UK, Vamos *et al.* [9] found that PCI rates increased significantly (IRR, 1.01, 95%CI, 1.005–1.03) in people with diabetes during 2004–2009. However, no studies have investigated national trends in the use and outcomes of PCI after AMI in diabetic patients in Spain.

In this study, we used national hospital discharge data to describe trends in the rate of AMI and use of PCI in patients with and without type 2 diabetes between 2001 and 2010 in Spain. In particular, we analyzed patient comorbidities and in-hospital outcomes such as length of stay and in-hospital mortality (IHM). Finally we analyzed the association between the use of PCI and IHM.

## Materials and Methods

### Ethics Statement

The Spanish National Hospital Database (CMBD) is hosted by the Ministry of Health Social Services and Equality (MSSSI). Researchers working in public and private institutions can request the databases by filling, signing and sending the questionnaire available the MSSSI web [10]. In the questionnaire the following information is required: 1. Researchers information. 2. Variables (years, diagnosis, procedures, outcomes and socio-demographic variables). 3. Objectives. 4. Analysis of patient records. 5. Proposed results dissemination. 6. Confidentiality Commitment.

All data used in this investigation was anonymized and de-identified by the MSSSI before it was provided to us.

Our investigation was presented and approved by the Institutional Review Board of the Rey Juan Carlos University.

According to the Confidentiality Commitment signed with the MSSSI we cannot provide anonymized or de-identified data to other researchers upon request. These researchers must request the data directly to the MSSSI.

### Design

We performed a retrospective, descriptive, epidemiology study using the CMBD, which compiles all public and private hospital data and therefore covers more than 95% of hospital discharges [11]. The CMBD is managed by the MSSSI and includes patient variables (sex, date of birth), date of admission, date of discharge, up to 14 discharge diagnoses, and up to 20 procedures performed during the admission. The MSSSI sets standards for registration and performs periodic audits [11].

We selected discharges for AMI in patients whose main medical diagnosis was classified according to the International Classification of Diseases-Ninth Revision, Clinical Modification (ICD-9-CM), codes 410.0–419.0. Discharge grouped by diabetes status as follows: no diabetes and type 2 diabetes (ICD-9-CM codes 250.x0 and 250.x2). Patients with type 1 diabetes were excluded (ICD-9-CM codes: 250.x1; 250.x3). PCIs were identified using the ICD-9-CM codes 00.66, 36.06, and 36.07.

We calculated the cumulative incidence of discharge rates after AMI for patients with type 2 diabetic and non-diabetes patients per 100,000 inhabitants. We also calculated the yearly age- and sex-specific cumulative incidence rates for diabetic and non-diabetic patients by dividing the number of cases by year, sex, and age group by the corresponding number of people in that population group according to data from the Spanish National Institute of Statistics, as reported at December 31 of each year [12].

The outcomes of interest included the proportion of patients who died during admission (IHM) and the mean length of hospital stay (LOS).

Clinical characteristics included information on overall comorbidity at the time of surgery, which was assessed by computing the Charlson comorbidity index (CCI). The index applies to 17 disease categories whose scores are totaled to obtain an overall score for each patient [13]. The index is subsequently categorized into three levels: 0, no disease; 1, one or two diseases; and 3, more than three diseases. To calculate the CCI we used 15 disease categories, excluding diabetes and AMI, as described by Thomsen RW *et al.* [14].

The percentage of use of PCI was calculated during the study period in patients with and without type 2 diabetes. We calculated LOS and IHM after PCI by diabetes status.

### Statistical Analysis

A descriptive statistical analysis was performed. Statistical significance was set at p<0.05 (2-tailed). In order to test the time trend in the use of PCI, we fitted separate Poisson regression models for patients with and without type 2 diabetes, using year of discharge, sex, age, and CCI as independent variables. For IHM, logistic regression analyses were performed with mortality as a binary outcome using the same variables for the group with and without diabetes and for the entire population. Statistical analyses were performed using Stata version 10.1 (Stata, College Station, Texas, USA).

## Results

During the 10-year study period, 513,517 discharges with AMI were identified. Patients with type 2 diabetes accounted for 30.3% of the total (155,676). Mean age was 67.26±13.95 years, and 60.5% were men. In patients without diabetes, the mean age was 71.38±11.18 years, and 73.2% were men (p<0.05).

Table 1 shows the annual hospital discharges rates for patients with and without type 2 diabetes. The cumulative incidence of discharges due to AMI in patients with diabetes increased from 56.3 cases per 100,000 inhabitants in 2001 to 71 cases per 100,000 inhabitants in 2004 and then decreased to 61.9 cases per 100,000 inhabitants in 2010. Cumulative incidence was significantly higher for men in both groups and in all the years studied.

**Table 1:** Hospital discharges due to acute myocardial infarction among patients with and without type 2 diabetes in Spain, 2001–2010.

	With Type 2 Diabetes				Without Diabetes			
Year	Total	Incidence	LOS (SD)	%IHM	Total	Incidence	LOS (SD)	% IHM
2001	12235	56.3	10.4(8.5)	13.2	34131	156.9	9.9(9.4)	11.2
2002	13864	62.9	10.6(9.1)	13.8	36904	167.5	9.8(9.6)	10.5
2003	15955	70.7	10.4(9.1)	12.9	36870	163.5	9.3(8.6)	10.3
2004	16396	71	10(8.3)	11.8	36550	158.3	9.1(10.3)	9.7
2005	16608	70.4	9.8(8.4)	12.1	36187	153.4	8.8(8.8)	9.2
2006	15754	65.4	9.6(8.7)	11.2	35566	147.5	8.5(8.4)	8.5
2007	16082	65.3	9.2(8.6)	11.0	35537	144.4	8.3(8.9)	8.5
2008	16221	64.6	9.2(8.3)	10.6	35799	142.5	8.1(8.7)	8.3
2009	16390	63.9	8.9(9.6)	9.8	35309	137.7	7.8(8.3)	7.9
2010	16171	61.9	8.6(9)	9.8	34988	133.8	7.7(9.5)	7.7
Total Men	94199	83.1	9.5(8.9)	9.4	262013	231.1	8.6(9.1)	7.4
Total Female	61477	50.1	9.9(8.6)	14.9	95828	78.1	9(9.1)	14.1
Total	155676	65.2	9.6(8.8)	11.5	357841	149.9	8.7(9.1)	9.2

Cumulative Incidence per100,000. Cumulative Incidence was calculated using the Spanish National Statistics Institute census projections [11]. LOS (SD): Mean length of stay (standard deviation). %IHM: In-Hospital Mortality.

The mean length of stay fell from 10.4 days in 2001 to 8.6 days in 2010 for patients with type 2 diabetes (p<0.05) and from 9.9 days in 2001 to 7.7 days in 2010 for patients without diabetes (p<0.05). LOS was significantly higher among men and women with than without diabetes in all the years analyzed (p<0.05).

Patients with type 2 diabetes had significantly higher IHM than patients without diabetes (11.5% vs. 9.2%). IHM decreased significantly from 13.2% in 2001 to 9.8% in 2010 among diabetic adults and from 11.2% to 7.7% among non-diabetic adults.

**Figure 1:**
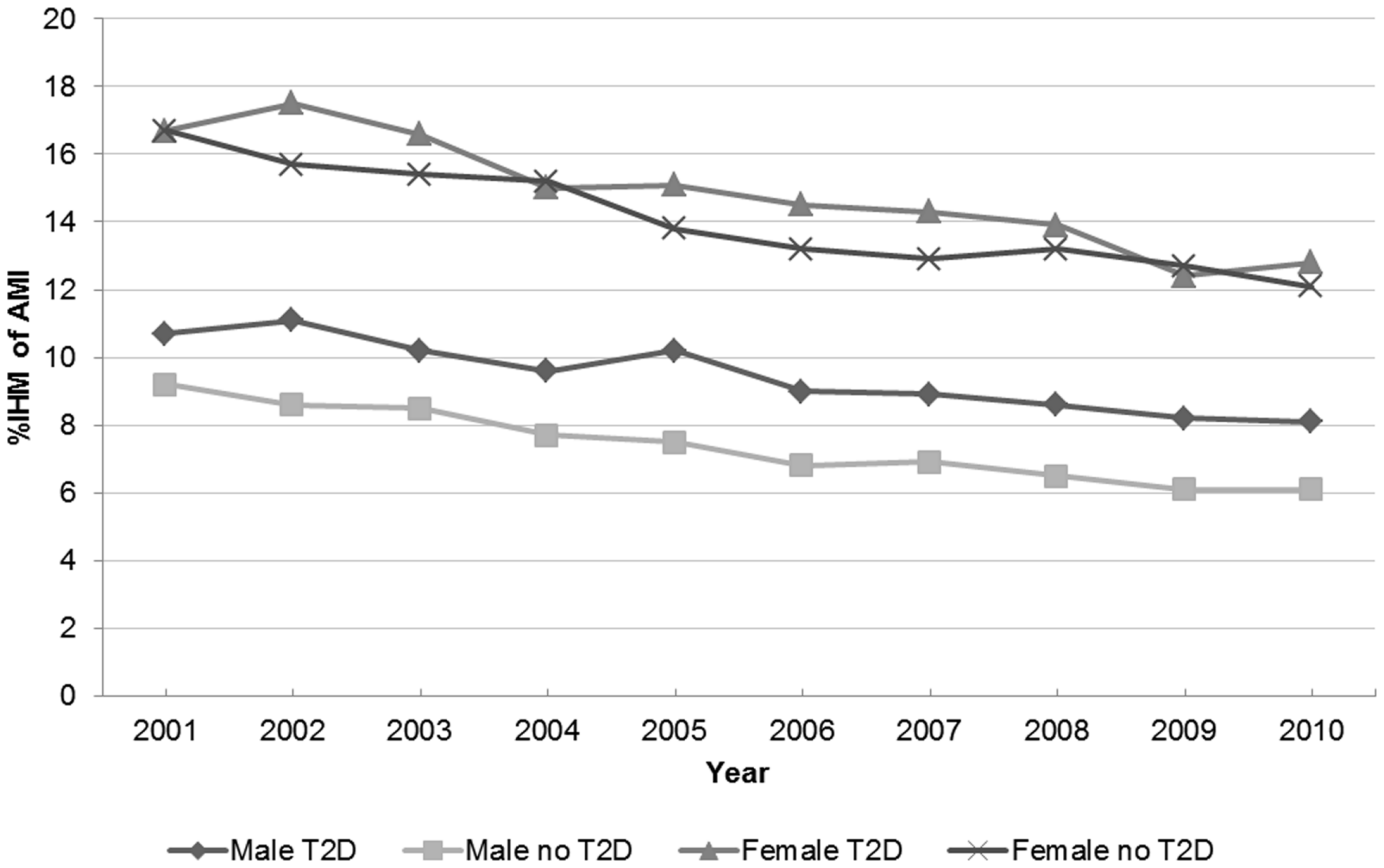
In-hospital mortality after AMI in patients with and without type 2 diabetes according to sex. IHM of AMI: In-hospital mortality after acute myocardial infarction. Male T2D: Men with type 2 diabetes. Male no T2D: Men without type 2 diabetes. Female T2D: Women with type 2 diabetes. Female without T2D: Women without type 2 diabetes.

IHM decreased for both sexes, although it was always greater in women with type 2 diabetes than in men with type 2 diabetes (Figure 1).

Table 2 presents the results of a multivariate analysis of the factors associated with cumulative incidence and IHM after AMI. When the year 2004 was used as the reference and after controlling for possible confounders, we observed that the cumulative incidence of discharges in patients with type 2 diabetes did not change significantly after this year (IRR, 0.98; 95%CI, 0.96–1.01).

**Table 2:** Multivariate analysis of the factors associated with cumulative incidence and in-hospital mortality after acute myocardial infarction in patients with and without type 2 diabetes in Spain, 2001–2010.

		With Type 2 Diabetes		Without Diabetes	
		Incidence (IRR)*	IHM (OR)†	Incidence (IRR)*	IHM (OR)†
Age (years)	35–60 years	1	1	1	1
	61–70 years	1.32 (1.30–1.34)	1.97 (1.82–2.13)	1.58 (1.54–1.61)	2.05 (1.95–2.16)
	71–80 years	2.11 (2.07–2.14)	3.46 (3.23–3.71)	1.23 (1.21–1.25)	3.99 (3.82–4.17)
	>80 years	1.21 (1.19–1.22)	5.84 (5.45–6.30)	1.72 (1.69–1.75)	7.79 (7.46–8.15)
Sex	Men	1	1	1	1
	Female	0.65 (0.64–0.66)	1.28 (1.24–1.32)	0.37 (0.36–0.38)	1.28 (1.25–1.32)
Charlson Index	0	1	1	1	1
	1–2	0.82 (0.81–0.83)	1.88 (1.82–1.95)	0.51 (0.50–0.53)	1.88 (1.83–1.92)
	≥3	0.20 (0.19–0.21)	2.64 (2.52–2.78)	0.09 (0.08–0.10)	2.76 (2.64–2.87)
Year	2001	0.75 (0.73–0.76)	1	0.93(0.92–0.95)	1
	2002	0.84(0.83–0.86)	1.03 (0.95–1.10)	1.01(0.99–1.02)	0.89 (0.85–0.94)
	2003	0.97(0.95–0.99)	0.90 (0.84–0.97)	1.00(0.99–1.02)	0.86 (0.82–0.90)
	2004	1	0.81 (0.76–0.87)	1	0.80 (0.76–0.84)
	2005	1.01(0.99–1.03)	0.82 (0.77–0.89)	0.99(0.97–1.00)	0.73 (0.70–0.77)
	2006	0.96(0.94–0.98)	0.76 (0.71–0.82)	0.97(0.96–0.98)	0.68 (0.65–0.72)
	2007	0.98(0.96–1.00)	0.73 (0.68–0.79)	0.97(0.96–0.98)	0.67 (0.64–0.71)
	2008	0.98(0.97–1.01)	0.69 (0.64–0.74)	0.98(0.96–0.99)	0.65 (0.62–0.68)
	2009	0.99(0.98–1.02)	0.63 (0.58–0.68)	0.97(0.95–0.98)	0.61 (0.58–0.65)
	2010	0.98 (0.96–1.01)	0.63 (0.58–0.68)	0.96(0.94–0.97)	0.61 (0.58–0.64)
PCI	Yes		1		1
	No		2,44 (2,32–2,56)		2,56 (2,42–2,66)

IHM: In-Hospital Mortality. PCI: Percutaneous Coronary Intervention.

Calculated using multivariate Poisson regression: Incidence Rate Ratios (IRR).

Calculate using logistic regression models: Odds Ratio (OR).

The logistic regression multivariate model and Poisson regression model were built using as dependent variables “death (yes/no)” and “Cumulative incidence of PCI” respectively, and as independent variables year, sex, Charlson comorbidity index, and age.

IHM was significantly greater in women with diabetes than in men with diabetes (OR, 1.28; 95%CI, 1.24–1.32) and in those with more diabetes-associated comorbidities (OR, 1.88; 95%CI, 1.82–1.95 [for those with 1 or 2 comorbidities] and OR, 2.64; 95%CI, 2.52–2.78 [for those with 3 or more comorbidities]). Those diabetic patients who did not receive a PCI had a 2.44-fold (95%CI, 2.32–256) higher probability of dying during their stay than those who underwent this procedure.

When we analyzed the entire database, patients with type 2 diabetes had significantly higher mortality than patients without diabetes after adjusting for age, gender, CCI, and year (OR, 1.14; 95%CI, 1.05–1.17).

### Coronary revascularization

Between 2001 and 2010, the overall number of PCIs in Spain was 168,537 (44,331 among patients with type 2 diabetes [26.3%]). There was a considerable male predominance in both patients with and patients without diabetes (70.0% and 81.2%, respectively). The mean age at the time of the PCI was significantly higher in patients with type 2 diabetes (67.2±0.05 years vs. 62.5±0.04 years).

Among those who underwent PCI, the mean LOS was significantly higher in patients with diabetes than in those without diabetes (9.31±0.04 days vs. 8.23±0.02 days). In addition, IHM was significantly higher in patients with diabetes (4.4% vs. 3.1%).

Patients with type 2 diabetes undergoing PCIs had a higher CCI than those without diabetes (39.2% vs. 28.5% with ≥1, respectively).

Table 3 shows the time trend for annual PCIs in patients with and without type 2 diabetes in Spain during 2001–2010. We found that use of PCI increased significantly in patients with and without diabetes. In 2001, 11.9% of patients with type 2 diabetes and 16.7% of patients without type 2 diabetes underwent PCI; in 2010, the corresponding figures were and 41.6% and 50.4%. The proportion of patients who had AMI and underwent PCI was significantly higher among those without diabetes in all the years studied.

**Table 3:** Characteristics and outcomes of hospital discharges after percutaneous coronary intervention among patients with and without type 2 diabetes in Spain, 2001–2010.

	2001	2002	2003	2004	2005	2006	2007	2008	2009	2010
Diabetes										
N*	1,467	2,206	2,885	3,640	4,439	4,781	5,474	6,067	6,645	6,727
%PCI*	11.9	15.9	18.1	22.2	26.7	30.3	34.1	37.4	40.5	41.6
Age, mean (SD)*	65.7	66.2	66.3	66.6	66.7	67.2	67.6	67.9	67.6	67.8
	(10.2)	(10.3)	(10.5)	(10.4)	(10.6)	(10.7)	(10.7)	(10.8)	(11.1)	(11.1)
Female, n (%)	457	702	861	1101	1304	1435	1691	1850	1980	1925
	(31.1)	(31.8)	(29.8)	(30.2)	(29.3)	(30.0)	(30.8)	(30.4)	(29.8)	(28.6)
CCI 0, n (%)	965	1420	1750	2226	2758	3086	3344	3524	3897	3994
	(65.7)	(64.3)	(60.6)	(61.1)	(62.1)	(64.5)	(61.1)	(58.1)	(58.6)	(59.3)
CCI 1–2, n (%)*	456	709	1004	1246	1442	1482	1846	2141	2265	2247
	(31.1)	(32.1)	(34.8)	(34.2)	(32.5)	(31.0)	(33.7)	(35.2)	(34.1)	(33.4)
CCI≥3, n (%)*	46	77	131	168	239	213	284	402	483	486
	(3.1)	(3.5)	(4.5)	(4.6)	(5.4)	(4.5)	(5.2)	(6.6)	(7.2)	(7.2)
LOS, mean (SE)*	11.3	10.9	10.5	9.8	9.7	9.3	9.1	9.0	8.6	8.2
	(0.23)	(0.21)	(0.18)	(0.13)	(0.12)	(0.12)	(0.11)	(0.1)	(0.1)	(0.1)
IHM, n (%)*	58	114	126	130	207	221	253	291	276	291
	(3.9)	(5.1)	(4.3)	(3.6)	(4.7)	(4.6)	(4.6)	(4.8)	(4.1)	(4.3)
No diabetes										
N*	5,715	7,624	8,882	10,252	12,249	13,216	14,807	16,325	17,499	17,637
%PCI*	16.7	20.6	24.1	28.1	33.8	37.1	41.6	45.6	49.5	50.4
Age, mean (SD)*	61.6	61.8	61.5	61.9	62.4	62.5	62.6	62.9	63.1	62.8
	(12.0)	(12.2)	(12.3)	(12.3)	(12.5)	(12.6)	(12.7)	(12.9)	(12.9)	(12.9)
Female, n (%)	1034	1363	1567	1808	2286	2526	2799	3115	3434	3473
	(18.0)	(17.8)	(17.6)	(17.6)	(18.6)	(19.1)	(18.9)	(19.0)	(19.6)	(19.6)
CCI 0, n (%)*	4188	5508	6367	7296	8675	9599	10548	11543	12438	12634
	(73.3)	(72.2)	(71.7)	(71.1)	(70.8)	(72.6)	(71.2)	(70.7)	(71.1)	(71.6)
CCI 1–2, n (%)*	1427	1968	2300	2688	3243	3262	3798	4208	4410	4339
	(24.9)	(25.8)	(25.9)	(26.2)	(26.4)	(24.6)	(25.6)	(25.7)	(25.2)	(24.6)
CCI≥3, n (%)*	100	148	215	268	331	355	461	574	651	664
	(1.7)	(1.9)	(2.4)	(2.6)	(2.7)	(2.6)	(3.1)	(3.5)	(3.7)	(3.7)
LOS, mean (SE)*	10.0	9.6	8.9	8.7	8.6	8.1	7.8	7.8	7.6	7.4
	(0.16)	(0.11)	(0.08)	(0.09)	(0.08)	(0.07)	(0.06)	(0.06)	(0.06)	(0.06)
IHM, n (%)*	227	238	297	324	379	367	422	497	566	531
	(3.9)	(3.1)	(3.3)	(3.1)	(3.0)	(2.7)	(2.8)	(3.0)	(3.2)	(3.0)

N:number of procedure; PCI:Percutaneous Coronary Intervention; SE:Standard Error;LOS:Length of stay; IHM:In-hospital mortality; CCI:Charlson comorbidity index;

p<0.05 Statistically significant differences were observed during 2001–2010.

As can be seen in Table 3, the mean age of a person with diabetes who underwent PCI was 65.7±10.2 years in 2001 and 67.8±11.1 years in 2010. The proportion of men varied from 68.9% in 2001 to 71.4% in 2010, and the prevalence of those with a CCI of ≥1 increased from 34.2% to 40.6% (p<0.05).

LOS after PCI decreased significantly during the study period in both groups of patients, showing higher values among those with diabetes in all the years analyzed (Table 3). IHM among those who underwent PCI decreased for patients without diabetes (3.9% to 3.0; p<0.05) but remained stable for those with diabetes (3.9% to 4.3%; p>0.05)

Multivariate analysis revealed that the cumulative incidence of discharge in patients with diabetes who underwent PCI increased significantly during the study period (IRR 3.49; 95%CI, 3.30–3.69) (Table 4).

**Table 4:** Multivariate analysis of the factors associated with cumulative incidence and mortality after percutaneous coronary intervention in patients with type 2 diabetes in Spain, 2001–2010.

		Incidence (IRR)*	In-hospital mortality (OR)†
Age (years)	35–60 years	1	1
	61–70 years	0.87 (0.85–0.89)	1.37 (1.16–1.61)
	71–80 years	0.70 (0.68–0.71)	2.56 (2.21–2.98)
	>80 years	0.33 (0.32–0.35)	3.31 (2.78–3.94)
Sex	Men	1	1
	Female	0.80 (0.79–0.82)	1.32 (1.20–1.46)
Charlson Index	0	1	1
	1–2	0.74 (0.73–0.76)	2.39(2.17–2.64)
	≥3	0.51 (0.49–0.53)	3.19 (2.73–3.73)
Year	2001	1	1
	2002	1.32 (1.24–1.41)	1.27 (0.92–1.76)
	2003	1.53 (1.43–1.62)	1.04 (0.76–1.43)
	2004	1.86 (1.75–1.98)	0.83 (0.60–1.14)
	2005	2.25 (2.12–2.39)	1.08 (0.80–1.46)
	2006	2.52 (2.38–2.67)	1.07 (0.80–1.45)
	2007	2.86 (2.70–3.03)	1.03 (0.77–1.38)
	2008	3.16 (2.98–3.34)	1.02 (0.75–1.36)
	2009	3.40 (3.21–3.60)	0.89 (0.66–1.19)
	2010	3.49 (3.30–3.69)	0.92 (0.69–1.23)

IRR: Incidence Rate Ratios calculated using multivariate Poisson regression.

OR: Odds Ratio calculated using logistic regression models.

The logistic regression multivariate model and Poisson regression model were built using as dependent variables “death (yes/no)” and “Cumulative incidence of PCI” respectively, and as independent variables year, sex, Charlson comorbidity index, and age.

After an adjusted multivariate analysis, the IHM among persons with diabetes who underwent a PCI did not change significantly over time. IHM was significantly greater in women than in men (OR 1.32; 95%CI, 1.20–1.46) and was higher in those with 1 or 2 diabetes-associated conditions (OR 2.39; 95%CI, 2.17–2.64) and ≥3 conditions (OR 3.19; 95%CI 2.73–3.73) than in those who had no associated comorbidities.

## Discussion

Our results reveal that more than 30% of Spanish adults who experience AMI have an associated diagnosis of diabetes. These results are consistent with those of Gore *et al.* (2012) [15], who showed that 29% of patients admitted to hospital for AMI in the US had diabetes.

From 2004 to 2010, rates of hospitalization for AMI in patients with type 2 diabetes decreased, but not significantly. The results of a study in the UK showed a considerable decline in hospital discharge for AMI in patients with diabetes between 2004–2005 and 2009–2010 (OR, 0.95; 95%CI, 0.93–0.97) [9]. Our results are consistent with this finding: rates of hospitalization for AMI increased initially before leveling off in 2004 and finally declining slowly from 71 cases per 100,000 inhabitants in 2004 to 61.9 cases per 100,000 inhabitants in 2010, thus revealing the same tendency as in the UK. The changes in these rates can be attributed to favorable trends in physical activity levels and cigarette smoking and increased use of effective treatments (eg, antihypertensive agents, ACE inhibitors, and lipid-lowering drugs) [9]. We think that the lack in improvement of lifestyles among diabetic patients [16], [17] and the absence of national prevention and treatment program throughout the study period may explain the different behavior in the reduction of hospitalizations for AMI between our data and those reported by Vamos et al [9].

IHM as a consequence of AMI decreased both in patients with and in patients without type 2 diabetes. Recent studies showed that patients with and without diabetes who have experienced AMI have lower mortality rates over time, suggesting that management of AMI patients has improved in recent years [9], [18]–[20]. More frequent and effective use of PCI, which reduced IHM in our study, has been observed by other investigators [18], [20]. We found that IHM for patients who did not receive a PCI was very similar in 2001 and 2010 for both those with diabetes (14.4% to 13.6%) and those without diabetes (12.6% to 12.4%).

Consistent with the results of other studies, and after adjusting for age and gender, we found that IHM for patients with AMI was significantly greater for patients with type 2 diabetes than for those without diabetes (11.5% vs. 9.2%) [21]–[23], possibly because these patients have a worse clinical status or are at a greater risk of complications [9], [18]. In our population, the proportion of patients with diabetes and a CCI≥3 was 10.0%, whereas the proportion for those without diabetes was 5.8% (p<0.05).

Our results are similar to those of studies reporting that women have a lower cumulative incidence of AMI than men [24], [25]. However, after controlling for possible confounders, we found that women with diabetes had significantly higher IHM rates than men with diabetes. These results are consistent with those of other studies that analyze differences in diabetes between the sexes [3], [24], [25]. A recent study indicated that women with diabetes have a greater risk of mortality than men (3.44; 95%CI, 2.47–4.79), especially when diagnosed at a later stage [26]. These data suggest that factors such as the extent of treatment and monitoring, underuse of medications recommended by clinical guidelines, and reduced efficacy of active agents may be more common in women with diabetes than in men with diabetes [27], [28].

### Coronary revascularization

During the study period, the number of PCIs performed in patients with type 2 diabetes increased considerably from 11.9% in 2001 to 41.6% in 2010. This result is consistent with those of other studies [9], [20], [29], in which PCI rates increased significantly owing to marked advances in stent technology and adjunctive pharmacology. One report documented the rapid progress in PCI treatment options for patients with diabetes and indicated that PCI devices (drug-eluting stents) were used more often in patients with severe comorbidities and multivessel disease and were associated with more frequent prescription of recommended cardiac medications at discharge [30].

Successful PCI has probably improved in-hospital survival rates. Therefore, IHM was more likely to be associated with patient clinical status and medical treatment strategy. Vamos *et al.* [9] found significant increases in IHM rates for PCI, despite technological advances in interventional techniques and improvements in periprocedural care. The authors explained their findings by referring to the increasing complexity of cases referred for PCI.

We found that IHM remained stable among diabetic patients with PCI. The higher comorbidity and older age can partially explain this lack of improvement.

In patients with AMI who had undergone PCI, women with type 2 diabetes had worse outcomes than men with diabetes. Our results are consistent with those of other studies, which suggest that the worse effect of diabetes on outcomes in women might be related to the onset mechanism of AMI, the success of the PCI procedure, and the higher burden of cardiovascular risk factors [20], [24], [31], [32].

The strength of our investigation lies in its large sample size and standardized methodology, which has previously been used to investigate diabetes in Spain and elsewhere [33], [34]. Nevertheless, our study is subject to a series of limitations. Our data source was the CMBD, an administrative database that contains discharge data for Spanish hospitalizations and uses information the physician has included in the discharge report; therefore, it does not include all the variables in the clinical history. Another limitation of this database is its anonymity (no identifying items such as number of the clinical history or the name of the hospital), which makes it impossible to detect whether the same patient was admitted more than once during the same year. In addition, patients who moved from one hospital to another would appear twice.

Nevertheless, this dataset, which was introduced in Spain in 1982, is a mandatory register, and its coverage is estimated to be more than 95% [10].

Unfortunately in Spain a validation study to assess the rate of unreported diagnosis of diabetes in administrative databases has not been conducted so far. However, a recent review and meta-analysis conducted by Leong A et al (2013) concluded that a commonly-used administrative database definition for diabetes had a pooled sensitivity of 82.3% (95%CI 75.8, 87.4) and specificity of 97.9% (95%CI 96.5, 98.8%), based on the findings of 6 studies with complete data available. While this definition appears to miss approximately one fifth of diabetes cases and wrongly classifies 2.1% of non-cases in the population as diabetes cases, it is likely sufficiently sensitive for monitoring prevalence trends in the general population if its accuracy remains reasonably stable over time [35].

We were unable to calculate diabetes-specific cumulative incidence rates, because no studies in Spain cover blood glucose measurements for the entire population; consequently, no precise estimation of the prevalence of diabetes is available [36]. Concerns have been raised about the accuracy of routinely collected datasets; however, these datasets are periodically audited. Consequently, the quality and validity of our dataset has been assessed and shown to be useful for health research [37].

In conclusion, we provide national data on changes in the burden of AMI events in Spain. Our results show that AMI hospitalization rates increased initially, before leveling off in 2004 and finally declining slowly in people with and without diabetes. Outcomes such as LOS and IHM are worse among persons with diabetes than without diabetes, although they improved over time for both groups. Higher comorbidity and female sex are associated with higher IHM.

The proportion of diabetic patients who undergo a PCI increased almost four-fold from 2001 to 2010. Older age and more comorbidity may explain why IHM among diabetic persons did not improve after a PCI during the study period.

Furthermore, given the rapid increase in prevalence of diabetes and the aging population, these findings emphasize the need for further improvement in the control of cardiovascular risk factors in people with diabetes.
